# Students’ academic engagement during COVID-19 times: a mixed-methods study into relatedness and loneliness during the pandemic

**DOI:** 10.3389/fpsyg.2023.1221003

**Published:** 2023-09-08

**Authors:** Laura Hendrick, Marie-Christine Opdenakker, Wander Van der Vaart

**Affiliations:** ^1^University of Humanistic Studies, Utrecht, Netherlands; ^2^Department of Teacher Education, Faculty of Behavioural and Social Sciences, University of Groningen, Groningen, Netherlands; ^3^Chair Group Education, University of Humanistic Studies, Utrecht, Netherlands; ^4^Chair Group Humanist Chaplaincy Studies for a Plural Society, University of Humanistic Studies, Utrecht, Netherlands

**Keywords:** basic psychological needs, self determination theory, online class, higher education, academic engagement, relatedness, loneliness

## Abstract

The COVID-19 outbreak forced higher education students to study online-only. Previous research indicates that forced solitude or loneliness can cause a variety of problems for students, among which is reduced academic engagement. The Basic Psychological Needs Theory, a sub-theory of Self-Determination Theory, relates academic engagement to three basic psychological needs (autonomy, competence and relatedness), whereas varying theories on loneliness highlight the complexities of engaging in a learning environment whilst feeling lonely. As university staff members have been struggling to keep students on task since the COVID-19 outbreak, the need arose for more knowledge about to what extent students have felt lonely, frustrated or satisfied in their need for relatedness and to what extent this affected their academic engagement. A convergent *Mixed Methods* research study was conducted among university students (*N* = 228) and an online questionnaire was administered to collect both quantitative and qualitative data. A series of multiple hierarchical regression analyses were performed, considering demographic characteristics, to analyze the quantitative data. Qualitative data was coded using a hybrid approach of deductive and inductive coding. Themes were generated that depicted in-depth issues of relatedness, loneliness, and academic engagement. Quantitative analysis demonstrated the importance for academic engagement of both (a) ‘basic need satisfaction and frustration’ of relatedness in life and in ‘social study context’, and (b) feeling (emotionally) lonely. The negative impact of frustration of relatedness seemed to be dominant but also overlapped with the effects of loneliness. The qualitative outcomes support and complement these quantitative results. The results showed that students’ academic engagement suffered from the loss of a shared physical space and from uncertainty about university policies. For a minority of students, however, the relief from social obligations that came along with social distancing was a blessing in disguise.

## 1. Introduction

Prior to the COVID-19 pandemic measures, it was self-evident that students and lecturers would regularly meet in a physically shared space (Labrague et al., 2021). However, due to the lockdown and social distancing policy, schools and universities were forced to rapidly adopt and pursue remote learning using video conferencing methods. For over a year, (higher education) students in the Netherlands and in other countries worldwide saw their teachers and fellow students for educational purposes online only (Benke et al., 2020). Similarly, the COVID-19 pandemic and measures (e.g., working and studying from home, obligation to go in quarantine when showing symptoms) impacted the schooling and life of all students worldwide (UNESCO’s Education Response to COVID-19, 2023).

Before the outbreak of COVID-19 and the resulting measures, it was unprecedented for an entire society to be banned from participating in joint activities (De Vos, 2020). Since 12 March 2020, the Netherlands was, as were many other countries worldwide around the same time, in the grip of the outbreak of COVID-19 and pursued a policy to lockdown educational institutions when necessary to mitigate the COVID-19 pandemic.

Early in the pandemic’s onset, research was conducted regarding effects of social distancing on students. Most of these studies focus so far on whether this situation makes students feel (more) lonely and in general, these studies indicate that this is the case (Benke et al., 2020; DUWO and Youngworks, 2020; Marchini et al., 2020; Labrague et al., 2021). Also, studies on effects of the lockdown and social distancing on students’ mental health were conducted indicating an increase in mental health problems (Sher, 2020; Raj and Bajaj, 2021; Dadhich et al., 2022; Rania et al., 2022). However, in contrast to the amount of these studies, at the start of the pandemic, little research has been done regarding the connection between the use of mandated virtual classrooms and students’ academic engagement (Müller et al., 2021). But as the lockdown continued, teachers, schools and universities experienced increased difficulties in keeping students on task and it became quite important to investigate what happened with students’ academic engagement. More specifically, it was relevant to explore factors that influenced or affected their engagement, since it is well known that academic engagement is core to the quality of learning, completion of learning tasks and performance (Fredricks et al., 2004; Klem and Connell, 2004; Martins et al., 2022). Furthermore, researchers stressed the importance to study academic engagement from the perspective of ensuring effective online teaching and student support (Bergdahl et al., 2020) during the pandemic (Gopinathan et al., 2022).

## 2. Theoretical background

### 2.1. Academic engagement

Academic engagement refers, among other things, to a student’s active involvement in tasks or activities related to school (Reeve, 2002, 2012) and includes students’ attention, interest, investment, and effort expenditure in the work of learning (Marks, 2000). It reflects a continuous and positive emotional state while learning and doing school tasks (Schaufeli et al., 2002). With *Academic engagement* we refer to positive participation, which can be noticed in observable ‘effort, attention and persistence during the initiation and execution of learning activities’ (Opdenakker, 2021, p. 5), and an ‘absence of disruptive behaviors’ (Martins et al., 2022, p. 805).

The concept of academic engagement has been extensively theorized in the framework of the Self-Determination theory (SDT henceforth), developed by psychologists Ryan and Deci (2018). This now established psychological motivation theory refers to three basic psychological needs and is continuously being developed to this day. The three psychological, presumed universal, basic needs (henceforth referred to as BPN) are autonomy, competence, and relatedness (*ibid.*). Contrary to binary theories on motivation and wellbeing, the SDT compartmentalizes motivation as a complex concept, albeit a continuum, with varying outcomes in behavior.

The SDT has its origin and basis in the human processes of motivation (*ibid.*) and suggests that people can become self-determined when the three BPN are satisfied (i.e.: autonomy, competence, and relatedness). Moreover, the SDT assumes that the fulfillment of these needs can be satisfied *or frustrated* by the social context, which seems very much applicable in the case of academic performance during a lockdown. The students were “forced” to study online, but also significantly less monitored during class by the authority of university staff. The lockdown created a new unprecedented educational context (Marchini et al., 2020). The theory provides a framework for examining the relationship between social context and motivation. The developers of the SDT argue that basic psychological needs must be ‘satisfied for psychological interest, development, and wellness to be sustained’ (Ryan and Deci, 2018, p. 10).

The basic psychological need for *autonomy* is ‘the need to self-regulate one’s experiences and actions’ (*ibid.*). The frustration of autonomy is experienced as ‘a sense of pressure and often conflict, such as feeling pushed in an unwanted direction’ (Vansteenkiste et al., 2020, p. 1). Being forced to have online classes only, may be an example of such a frustrating experience. The need to feel *competent* emerges as an inherent striving and curiosity (Ryan and Deci, 2018, p. 10). As Vansteenkiste et al. (2020) state: ‘It becomes satisfied as one capably engages in activities and experiences opportunities for using and extending skills and expertise’ (Vansteenkiste et al., 2020, p. 1). The *frustration* of competence, in a social context, leads to experiences of ‘failure and helplessness’ (*ibid.*). The more satisfied the BPN are, the more engaged the individual will behave, which indicates a healthy, sustainable motivation (Ryan and Deci, 2018). The often-last-mentioned need, *relatedness,* ‘*denotes the experience of warmth, bonding, and care, and is satisfied by connecting to and feeling significant to others*’ (Vansteenkiste et al., 2020, p. 1). The *frustration* of relatedness ‘comes with a sense of social alienation, exclusion, and loneliness’ (Vansteenkiste et al., 2020, p. 1). “Feeling lonely” *indicates* frustrated relatedness, although it is not a BPN *per se* (Baumeister and Leary, 1995; Vansteenkiste et al., 2020).

### 2.2. Relatedness and loneliness

In the current study, the influence of relatedness and loneliness on academic engagement is investigated, whilst also looking at how both influences relate to each other. That in-person meetings were prohibited, and might be again in the future, focuses this study on the variations in ‘relatedness’ and ‘loneliness’, with ‘relatedness’ investigated more in-depth than ‘loneliness’ (Kiltz et al., 2023), even though we concur that ‘autonomy’ and ‘competence’ are equally important in SDT.

Relatedness as a *need* works two ways: receiving and *being able* to give love, intimacy, and companionship. This means that beside feeling related because people seem to care for you, you need to be satisfied in your social context to care for others as well (Ryan and Deci, 2018). People feel related *‘by being significant members’* (Ryan and Deci, 2018, p. 11) of a community. Their sense of relatedness is frustrated when they feel their social context communicates their insignificance as a member on an individual and/or community level. This social context can be any gathering of people joined by a shared objective, such as a household, a workplace, and – considering this study – an educational context such as a university or college.

The frustrated need of relatedness is conceptually close to ‘loneliness’ (Chen et al., 2015; Saricali and Guler, 2022), which is another concept that is examined in this study. ‘Loneliness’ is a widely and extensively researched subject (Yanguas et al., 2018). Sociologist De Jong-Gierveld defines loneliness as ‘the negative outcome of a cognitive evaluation of a discrepancy between (the quality and quantity of) existing relationships and relationship standards’ (De Jong-Gierveld et al., 2006, p. 495). This means that loneliness is experienced when the relatedness to others seems to fall short in comparison to what is *expected* of feeling related. Following the work of Weiss (Russell et al., 1984), De Jong-Gierveld distinguishes between *social* and *emotional* loneliness (De Jong-Gierveld et al., 1999). Social loneliness refers to the lack of social integration in a group, whilst emotional loneliness points to the lack of a personal, intense, deep connection to *one* other person. Neurologists Cacioppo and Patrick define loneliness as an alarm. This alarm signals our ancient, prehistoric instincts that something is terribly, dangerously off: *‘[…] evolution fashioned us not only to feel good when connected, but to feel secure […] evolution not only shaped us to feel bad in isolation, but to feel insecure, as in physically threatened’* (Cacioppo and Patrick, 2009, p. 15). According to economist Hertz, loneliness not only makes us feel uncared for and unloved by the individuals we meet in our daily lives, but it also makes us:

feel unsatisfied and uncared for […] by our community, our government. It’s about feeling disconnected […] also from ourselves. […] includes but is also greater than our desire to feel close to others because it is also a manifestation of our need to be heard, to be seen, to be cared for, to have agency, to be treated fairly, kindly and with respect. (Hertz, 2021, pp. 8–9).

The above theories endorse various life conditions that may alleviate loneliness: not only feeling part of a community, or having an intense relationship, but also humans’ need to feel they “exist” in the social realm by what is called ‘social mini-interactions’ (Cacioppo and Patrick, 2009; Sandstrom and Dunn, 2013, 2014; Hertz, 2021).

Studies regarding the effect of COVID-19 measures on student’s psychological wellbeing indicate that loneliness among students was prevalent (Li and Xu, 2020; Rania et al., 2022), albeit not as clear-cut as was expected (Phillips et al., 2022). Students experienced “waves” of loneliness, which means that it was not a linear experience of becoming lonelier. Also, their experience of loneliness depended on whether they felt lonely on campus prior to the pandemic, in which case sometimes the measures were even considered a relief, since these students were no longer confronted with their loneliness daily during lonely lunches or the absence of invitations to social gatherings (*ibid.*). Now everyone was equally alone in their dorm or apartment (Rania et al., 2022). The experience of loneliness is clear, yet thus connected to circumstance that it is difficult to pinpoint. The COVID-19 measures provided an accidental social experiment in which researchers could assess certain theories on, for example, motivation, academic engagement, (and their relationship with) basic need satisfaction, relatedness, and loneliness.

The unprecedented online-only educational context demanded communicational skills in the digital world to feel connection (Chiarchiaro et al., 2021). Prior to the pandemic, researchers found that despite the growing number of online educational classes, digital communication was still poor compared to face-to-face communication (Treve, 2021). The sudden change in the way of communicating between students, faculty staff and teachers might have led to increased feelings of loneliness, as the connection between loneliness and unclear, uncomfortable communication is deemed apparent by researchers in the past (Zakahi and Duran, 1982) and more present (Yuldashev et al., 2022).

## 3. Aim of the study and expectations

The main aim of this study is to investigate the effects of the prolonged lockdown on students’ academic engagement and to explore the factors that are expected to influence these effects. Considering the unprecedented situation of social distancing since 2020, the BPN ‘relatedness’ and the conceptually close concept of ‘loneliness’ are expected to be important, since research on SDT has already found the satisfaction and frustration of the other two BPN (i.e.: autonomy and competence) to be salient in affecting academic engagement in higher education students. Moreover, SDT assumes - and research on students’ academic engagement indicates - that all three basic psychological needs are important regarding academic engagement.

It is important to examine when people feel related or lonely in relation to academic engagement since global outbreaks are still ongoing (WHO Coronavirus (COVID-19) dashboard, n.d.). Furthermore, the possibility of future lockdowns is implicated even now that the WHO has downgraded the pandemic (Euronews, 2023). By focusing on (individual) students’ experiences with feeling related and lonely during the COVID-19 lockdown in life in general and in relation to their school’s social (study) context and by examining the effects of these experiences on their academic engagement, we aim to contribute to a deeper insight into the different needs (individual) students have in general and, in particular, in relation to their schools’ social context relevant for their academic engagement during times of prolonged lockdowns characterized by social distancing. A more in-depth knowledge on this will also be helpful for educational institutions to optimize their policies and communication with students in times of future lockdowns.

To address our aims, we formulated the following main question: Did experiences of loneliness and relatedness in life and in school social study context (‘relatedness in study’ henceforth) affect students’ academic engagement during COVID-19 lockdown, and if so to what extent?

To answer this main question, this study will focus on answering two quantitative questions and one qualitative question, namely:

1. How did students score and differ on academic engagement, relatedness, and loneliness during the COVID-19 lockdown?
2. What was the impact of BPN satisfaction and frustration of relatedness and experiences of loneliness on students’ academic engagement?
3. Which experiences of relatedness and loneliness did students express in relation to their academic engagement?

The qualitative question focuses on in-depth data about the students’ experiences and aims to supplement the first two quantitative, more generalized results. The results will be discussed in the light of existing literature on COVID-19 measures and their effects on mental health in education, as well as considering existing theories on loneliness.

## 4. Materials and methods

To answer the main question, a convergent mixed-methods approach was applied (Creswell, 2014). This particular method has also been used in the context of COVID-19 related studies (LoGiudice and Bartos, 2021). The rationale for utilizing Mixed Methods (MM henceforth) is to explore complex emotionally driven behavior (e.g., engagement, withdrawal) as described by SDT-theorists from multiple perspectives, seeking to find underlying mechanisms.

### 4.1. Participants and procedure

Participants were all students of a small independent, government-funded university in the centre of the Netherlands which is known for its focus on interpersonal contact between peer students and between teaching staff and students. The university offers Bachelor and (Pre-) Master degrees and a PhD/graduate school program in the domains of the humanities and social sciences. A pre-master is a specific educational pathway to bridge the gap between educational/discipline levels.

Respecting the COVID-19 measures at the time, an online questionnaire with closed (quantitative data) and open-end questions (qualitative data) concerning relatedness, loneliness and academic engagement was administered to the students. It included also questions concerning demographic factors that are of relevance to academic engagement, relatedness and/or loneliness and enables to characterize the population on relevant demographics. This survey was administered once and could be filled in from September until late October 2020. Students were approached through the university communication channels and informed consents were obtained before students started with filling in the questionnaire. Two hundred and twenty-eight students from the 611 enrolled students completed the survey. This corresponds to a response rate of 37.3% which is generally expected for online studies and deemed sufficient (Daikeler et al., 2021). A comparison between these students and the student population of the university at the time of measurement revealed that this sample group represents the population of enrolled students quite well, regarding age, study programme and gender. 57.0% of the respondents were 18–25 years old, 20.2% between 26–33 years, 5.7% between 34–41, 3.5% between 42–49, 4.8% between 50–57 and 0.9% between 58–65 years old (7.9% missing). 75.0% were female students, 17.1% were male (7.9% missing). 41.2% were Bachelor students, 40.8% master students and 11.0% pre-master students (7% missing). 75.9% of the respondents was living with others and 15.4% was living alone (8.8% missing). More detailed information on the group of respondents (e.g., crossed and nested tables) and the student population can be found in Supplementary material. We adjusted the questionnaire for the COVID-19 context by introducing a brief text before each set of questions, guiding respondents to consider their answers within the framework of the COVID-19 situation.

### 4.2. Instruments and analysis – quantitative part of the study

#### 4.2.1. Instruments

##### 4.2.1.1. Academic engagement

Academic engagement is measured by students’ self-report and refers to engaged behavior (and emotion; see also Opdenakker, 2021). It is based on a frequently used scale in Dutch scientific research based on the work of Roede (1989) and is in line with the concept of (behavioral) engagement of Skinner et al. (2008, 2009). Examples of items are: ‘I actively participate in the (online) educational activities (lectures/seminars)’ and ‘I can easily focus while studying’. Items were scored on a five-point Likert scale ranging from 1 = ‘Completely disagree’ to 5 = ‘Completely agree’. The scale consisted of five items and the reliability of the scale scores is 0.80 (Cronbach’s α), 0.81 (McDonald’s ɷ) and 0.82 (Guttman Lambda_2_).

##### 4.2.1.2. Relatedness satisfaction/frustration in life in general (‘relatedness in life’)

Students were asked to score items that measure both satisfaction and frustration of BPN *Relatedness* regarding their lives in general since the onset of the COVID-19 measures. An example of satisfaction of relatedness in life is ‘I feel that the people I care about also care about me’, whereas an example of a frustration in relatedness in life is ‘I feel excluded from the group I want to belong to’. Items were scored on a five-point Likert scale (1 = ‘Completely disagree’ to 5 = ‘Completely agree’). The questions are based on ‘The Basic Psychological Need Satisfaction and Frustration Scale’ (BPNSFS) (Chen et al., 2015; Cardella et al., 2020; Ryan and Deci, 2020). ‘The satisfaction of relatedness in general’ subscale consisted of four items (Cronbach’s α and Guttman Lambda_2_ = 0.87, McDonald’s ɷ = 0.88) and the ‘frustration of relatedness in general’ subscale consisted of four items (Cronbach’s α, Guttman Lambda_2_ and McDonald’s ɷ = 0.69). The relatedness scales were validated in previous studies with comparable Dutch-speaking student groups in Belgium (e.g., Chen et al., 2015) and the Netherlands (e.g., Kiltz et al., 2023).

##### 4.2.1.3. Relatedness satisfaction/frustration in school social study context (‘relatedness in study’)

Consequently, students were asked to score items indicating their satisfaction (2 items) and their frustration (2 items) of their *Relatedness regarding their study and learning environment* since the onset of the COVID-19 measures. An example of satisfaction of relatedness in study is ‘I feel connected to my friends and fellow students on the university’, whereas an example of a frustration in relatedness in study is ‘I feel that teachers and fellow students are cold and distant towards me’. Items are based on the BPNSFS scale (*ibid.*) but focus now on the relatedness dimension in students’ educational social study context. These items are added to assess the difference in *relatedness satisfaction/frustration* between life in general and life as a student. The reliability of the scale scores were, respectively, 0.61 (Cronbach’s α and Guttman Lambda_2_) for ‘satisfaction of relatedness in study’ and 0.57^1^ (Cronbach’s α and Guttman Lambda_2_) for the ‘frustration of relatedness in study’ subscales.

##### 4.2.1.4. Social and emotional loneliness

The De Jong-Gierveld scale (DJGS), which was originally developed in Dutch but is nowadays also used worldwide in studies with adults and college/university students (De Jong-Gierveld et al., 2010; Uysal-Bozkir et al., 2015), was used to assess loneliness because it can measure social and emotional loneliness separately (De Jong-Gierveld and Van Tilburg, 1999; De Jong-Gierveld et al., 2006; De Jong-Gierveld and Van Tilburg, 2010). Previous studies have demonstrated its reliability and validity (De Jong-Gierveld and Van Tilburg, 1999; De Jong-Gierveld et al., 2006; De Jong-Gierveld and Van Tilburg, 2010) and more recent studies regarding COVID-19 (Heidinger & Richter, 2020; Menze et al., 2022).

Social loneliness refers to the extent to which students indicate that they experience a lack of social integration in a community or of belonging to (a) friends (group) and being able to fall back on this. Emotional loneliness refers to the extent to which students lack an intense emotional connection, a close, intimate bond with one or more persons.

An example of social loneliness is ‘There are plenty of people I can lean on when I have problems’ (items were recoded), whereas an example of emotional loneliness is ‘I experience a general sense of emptiness’. Normally speaking, these questions are referring to loneliness in general.

However, since we were interested in students’ feelings of loneliness since the social distancing measures, we introduced the questions with referring to the period from March 12, 2020 (initiation government induced social distancing measures in the Netherlands). Items were scored on a five-point Likert scale (1 = ‘Completely disagree’ to 5 = ‘Completely agree’), with a higher score indicating more loneliness. The ‘social loneliness’ subscale consisted of five items (Cronbach’s α. Guttman Lambda_2_ and McDonald’s ɷ = 0.87), and the ‘emotional loneliness’ subscale consisted of six items (Cronbach’s α, Guttman Lambda_2_ and McDonald’s ɷ = 0.83).

To investigate the validity and validation of the scales for our student group further, confirmatory factor analyses were carried out for the academic engagement scale, the two scales of relatedness in life in general, the two scales of relatedness in school social study context, and the two loneliness scales with LISREL 8.80 (Jöreskog and Sörbom, 2006; Kumalasari and Priharsari, 2023). Overall, the variety of model fit indices indicated an acceptable to good fit according to the cutoff criteria for fit of Hu and Bentler (1999). (Detailed results of the analyses are available from the authors on request).

##### 4.2.1.5. Control variables

Because there are students of different age groups and it can be assumed that they are therefore in different life phases (Jiménez Rodrigo and Márquez Lepe, 2014), questions about age, living situation, gender, and study phase (Bachelor, Premaster, Master) were included as control variables Age is categorized into six groups for descriptive analysis and measured in years for regression. Gender is coded as 0 for male and 1 for female, while life situation is coded as 0 for cohabiting and 1 for living alone. Study phase 1 (Dummy 1) compares the Bachelor group (coded as 1) to other groups (coded as 0), and Study phase 2 (Dummy 2) compares the Master group (coded as 1) to other groups (coded as 0).

#### 4.2.2. Analysis

First, descriptives (i.e., mean and standard deviation) on the scales were calculated and means were compared using *paired t-tests (to answer sub-question one)*.

Second, to see what the impact of BPN satisfaction and frustration of relatedness and experiences of loneliness on students’ academic engagement was (sub-question two), a series of multiple hierarchical regression analyses were performed, considering the demographic characteristics (covariates/controls) and the independent variables. First, we explored whether there were strong (significant) correlations between the (independent) variables. This turned out to be the case (Table 1).

**Table 1:** Pearson correlations between relatedness, loneliness and academic engagement scales (*N* = 151–198).

	1	2	3	4	5	6	7
(1) Acad. engagement	–						
(2) Satisf. related life	0.242^**^	–					
(3) Frustr. related life	−0.284^**^	−0.735^**^	–				
(4) Satisf. related study	0.206^**^	0.268^**^	−0.294^**^	–			
(5) Frustr. related study	−0.292^**^	−0.284^**^	0.439^**^	−0.516^**^	–		
(6) Social loneliness	−0.187^*^	−0.674^**^	0.616^**^	−0.240^**^	0.368^**^	–	
(7) Emotional loneliness	−0.316^**^	−0.473^**^	0.503^**^	−0.312^**^	0.470^**^	0.477^**^	–

*Correlation is significant at the 0.05 level (2-tailed); **Correlation is significant at the 0.01 level (2-tailed).

We see a moderate to strong correlation between the satisfaction and frustration scales, but the correlation is clearly stronger between the general relatedness scales than between the academic relatedness scales. Social and emotional loneliness are moderately related, and here is also a moderate correlation between the general relatedness scales and the loneliness scales. The strongest correlation is seen between the scales ‘frustration’ and ‘satisfaction of general relatedness’ (*r* = 0.735, *p* = 0.01). This is the only correlation value within the “strong” category, according to Dancey and Reidy (2011) variables with such strong correlations should not be included together in a regression analysis for reasons of multicollinearity (Tabachnick et al., 2013). To keep the VIF (Variation Inflation Factor) below ‘5’, all possible predictor variables are centered around 0, by subtracting the mean. These centered variables are included in the final regressions. Because of the moderate to strong correlations between independent covariate variables, it was decided to add independent variables separately to Model 1 (=model with demographics) to see whether they were significant. If so, they were added to Model 2. In the first series of analyses, the relatedness scales were introduced first in addition to the demographic covariates (Model 2). In a second series of analysis, the loneliness scales were introduced first (Model 2). Finally, both were added to Model 1 (Model 3).

### 4.3. Data collection coding and analysis – qualitative part of the study

#### 4.3.1. Data collection

Qualitative data was collected using open-ended questions within the same online survey that collected the quantitative data. The main aim was to find additional aspects or perceptions that might help interpret the quantitative outcomes. At the end of the survey four open-ended (no word limit to encourage elaboration) questions were asked as to elicit written experiences on how related, lonely, and academically engaged students felt in the context of the COVID-19 lockdown, if at all.

The questions read as follows:

- ‘To what extent have the measures taken in response to COVID-19 affected your life?’
- ‘To what extent do you feel limited in your personal freedoms and to what extent do you feel inclined to participate in social distancing?’
- ‘What are your expectations for your future and/or your remaining time as a student?’
- ‘Is there anything else you would like to say, issues that have not been addressed in the questionnaire and which you consider relevant for this research?’

Respondents answered the open-ended questions extensively and indicated they appreciated to have someone ask them about how they were doing during the COVID-19 lockdown. Some compared answering these questions to writing in a diary.

To find out whether students who *did* answer the open, qualitative questions (*N* = 123) significantly differed in characteristics (for representation) from the rest of the respondents (*N* = 105), *Chi-square* tests were performed on the covariates referring to student (background) characteristics. These tests showed that there is no significant difference between the group of respondents who did and those who did not participate in the open questions regarding these covariates. This means that age (*χ*^2^(5) = 4.46, *p* = 0.49), gender (*χ*^2^(1) = 3.59, *p* = 0.06), living situation (*χ*^2^(1) = 0.24, *p* = 0.62) and study phase (*χ*^2^(2) = 5.23, *p* = 0.07) were equally divided in both groups. *In the same line independent samples t-tests* with Bonferroni correction showed no differences between both groups regarding the dependent variable of academic engagement, the relatedness variables, and the loneliness variables: Academic engagement (*t*(179) = −0.20, *p* = 0.85), satisfaction relatedness life (*t*(149) = −0.64, *p* = 0.52), frustration relatedness life (*t*(149) = 0.15, *p* = 0.88), satisfaction relatedness study (*t*(154) = 0.48, *p* = 0.63), frustration relatedness study (*t*(154) = −0.25, *p* = 0.80), social loneliness (*t*(196) = 0.97, *p* = 0.34), and emotional loneliness (*t*(196) = 0.20, *p* = 0.84).

#### 4.3.2. Coding

A hybrid approach of deductive and inductive coding was applied (Fereday and Muir-Cochrane, 2006; Swain, 2018). First, the qualitative data (i.e.: all essay responses from 123 respondents) were converted from *Qualtrics* into *Excel*. In *Excel*, the documents were divided into groups according to age, gender, living situation and stage of study. The documents were then analysed using *Atlas.TI*, a widely used programme for the thematic segmentation of texts (Soratto et al., 2020). Initially, based on our problem statement, texts were open coded in respondents’ own words (e.g.: ‘I feel abandoned’, ‘I have more time for study’, etc.) resulting in 300 codes. These codes were categorized into primary code groups defined by the main concepts (i.e.: academic engagement; satisfaction and frustration of BPN relatedness; loneliness) as discussed in ‘Theoretical background’. That is, into codes such as ‘satisfaction relatedness’, ‘loneliness’, etc. Finally, by finding patterns and relationships between codes, axial codes were generated that formed the main themes of the substantive analysis (*Ibid.*; Vollstedt and Rezat, 2019; Table 2).

**Table 2:** Shortened display of the coding scheme.

Open-codes	Deductive code groups	Axial codes
More time for study; I’m better able to concentrate; Looking forward to the future…	Academic engagement [[…] positive participation, which can be noticed in observable ‘effort, attention and persistence during the initiation and execution of learning activities’ (Opdenakker, 2021, p. 5)].	Increased ‘mindspace’ for studies; Less distraction from peers and social mini-interactions; More in tune with ‘self’; Less worry peripheral matters.
Study no longer fun; I’m behind schedule; The lecturer is droning on; I miss a proper place to study; I do not understand the material…	Academic withdrawal (indicating lack of positive participation: i.e.: *struggling* with focus, attention and persistence ‘during the initiation and execution of learning activities’).	Disliking home study; Whom to turn to; Lower quality study; Lack of discussing material.
I’m happy: family quality time; Walking with housemates is pleasant; I see who my real friends are…	Satisfaction relatedness [Receiving and *being able* to give love, intimacy, and companionship (Ryan and Deci, 2018)].	More space for family; Less worry peripheral matters; More energy for close environment.
No one will ask for you if you do not show up online; Teachers are supposed to care about their students….…	Frustration relatedness [Comes with a sense of social alienation, exclusion, and loneliness’ (Vansteenkiste et al., 2020, p. 1)].	Community loss; Lack of discussing material; Whom to turn to; Lack of communication from university.
I feel abandoned; I’d think the university would care about students; I’m not hearing any sound from uni…	More loneliness [‘The negative outcome of a cognitive evaluation of a discrepancy between (the quality and quantity of) existing relationships and relationship standards’ (De Jong-Gierveld et al., 2006, p. 495)].	Not belonging as expected; Community loss; Community does not care; Feeling unworthy; Lack of communication from university.
I have enough people to talk to; I just like to do things by myself.	Less loneliness (Positive outcome of the above mentioned relationship between ‘existing relationships and relationship standards’)	Stable connections, despite social distancing.

#### 4.3.3. Analysis

The themes generated by the axial codes were examined more closely to see how, for example, connectedness and loneliness in the qualitative data are related to academic commitment and how this might elaborate the quantitative results. Being a cross-sectional study, the quantitative results cannot fully determine whether the variables were affected by the COVID-19 lockdown and the qualitative analysis serves to provide that information.

Table 2 indicates that certain themes show overlap, which was expected from the theory (e.g.: “Feeling lonely” *indicates* frustrated relatedness, although it is not a BPN *per se* Baumeister and Leary, 1995; Vansteenkiste et al., 2020). These overlapping themes serve to find nuances in the experiences of feeling related or lonely, as the theory points out that these concepts are complex, and subtleties might get lost in the quantitative results.

To further clarify the quantitative results, we looked at what stood out from the data and what had not been anticipated as a theme from the theory, such as the experienced lack of communication from the university.

## 5. Results

### 5.1. How did students score and differ on academic engagement, relatedness, and loneliness during the COVID-19 lockdown?

#### 5.1.1. Academic engagement

The average score on academic engagement during COVID-19 was 3.17 (*SD* = 0.75), indicating that students felt generally neutral towards positive regarding their academic engagement during COVID-19 times (Table 3).

**Table 3:** Descriptive statistics of the variables referring to relatedness, loneliness and academic engagement.

	*N*	M	SD
Academic engagement	181	3.17	0.75
Satisfaction relatedness life	151	4.17	0.72
Frustration relatedness life	151	1.88	0.69
Satisfaction relatedness study	156	2.89	0.89
Frustration relatedness study	156	2.63	0.95
Social loneliness	198	2.05	0.78
Emotional loneliness	198	3.15	0.88

#### 5.1.2. Relatedness in life and study

Regarding relatedness in life, the average score for students’ satisfaction was relatively high (*M* = 4.17, *SD* = 0.72) and for students’ frustration relatively low (*M* = 1.88, *SD* = 0.69) (Table 3).

However, we see a more negative score when it comes to relatedness in study with an average score on satisfaction of 2.89 (*SD* = 0.89). Frustration of students’ relatedness in study was relatively high 2.63 (*SD* = 0.94) compared to their frustration of relatedness in study (Table 3).

The results of a *paired t-test* show that students’ relatedness satisfaction is significantly lower regarding their academic environment (*t*(150) = 15.87, *p* = 0.000) compared to their satisfaction in life in general. Also, students feel significantly more frustrated in their need for relatedness in their academic environment compared to this need in life in general (*t*(150) = −10.10, *p* = 0.000).

#### 5.1.3. Social and emotional loneliness

Regarding social loneliness, the average score was relatively low (*M* = 2.05, *SD* = 0.78) and students seemed to score higher on emotional loneliness (*M* = 3.15, *SD* = 0.88; Table 3). This difference was found to be significant (*t*(197) = −18.15, *p* < 0.001) indicating that students, on average, missed more an intense deep connection than integration in a community.

### 5.2. What was the impact of BPN satisfaction and frustration of relatedness and experiences of loneliness on students’ academic engagement?

#### 5.2.1. Impact on academic engagement

Results of the hierarchical multiple regression analyses can be found in Tables 4, 5. In the first series of analyses (see Table 4), the relatedness scales were introduced first in addition to the demographic covariates. In a second series of analyses, the loneliness scales were introduced first (see Table 5).

**Table 4:** Results of the hierarchical regression analysis I (with relatedness scales included before loneliness scales).

Predictors	Model 1			Model 2			Model 3		
	B	SE	*t*	B	SE	*t*	B	SE	*t*
Age	0.01	0.01	1.55	0.01	0.01	0.93	0.01	0.01	0.98
Gender	−0.31	0.17	−1.84	−0.29	0.16	−1.84	−0.27	0.16	−1.65
Living situation	−0.12	0.16	−0.72	−0.02	0.16	−0.13	−0.01	0.16	−0.05
Study phase dummy 1	−0.39	0.25	−1.59	−0.48	0.24	−2.02*	−0.42	0.24	−1.71
Study phase dummy 2	−0.18	0.22	−0.82	−0.30	0.22	−1.40	−0.29	0.22	−1.33
Frustration relatedness study				−0.16	0.07	−2.19*	−0.12	0.08	−1.61
Frustration relatedness life				−0.21	0.10	−2.10*	−0.16	0.12	−1.35
Social loneliness							0.00	0.11	0.04
Emotional loneliness							−0.12	0.09	−1.33
*R* ^2^	0.10			0.19			0.20		
F for *R*^2^ change	2.91*			8.06**			0.92		

*Significant at the 0.05 level; **significant at the 0.01 level.

**Table 5:** Results of the hierarchical regression II (with loneliness scales included before relatedness scales).

Predictors	Model 1			Model 2			Model 3		
	B	SE	*t*	B	SE	*t*	B	SE	*t*
Age	0.01	0.01	1.55	0.01	0.01	1.57	0.01	0.01	0.98
Gender	−0.31	0.17	−1.84	−0.26	0.16	−1.58	−0.27	0.16	−1.65
Living situation	−0.12	0.16	−0.72	−0.02	0.16	−0.11	−0.01	0.16	−0.05
Study phase dummy 1	−0.39	0.25	−1.59	−0.29	0.24	−1.22	−0.42	0.24	−1.71
Study phase dummy 2	−0.18	0.22	−0.82	−0.23	0.22	−1.04	−0.29	0.22	−1.33
Social loneliness				−0.10	0.10	−1.05	0.00	0.11	0.04
Emotional loneliness				−0.21	0.08	−2.47*	−0.12	0.09	−1.33
Frustration relatedness study							−0.12	0.08	−1.61
Frustration relatedness life							−0.16	0.12	−1.35
*R* ^2^	0.10			0.17			0.20		
F for *R*^2^ change	2.91*			6.07*			2.74		

*Significant at the 0.05 level; **significant at the 0.01 level.

As can be seen in Table 4, Model 1, the model including only the demographic covariates, was found to be statistically significant, *F*(5,138) = 2.91, *p* < 0.05, and explained 10% of the variance in academic engagement (*R^2^* = 0.10). However, on closer inspection of Model 1, no unique demographic predictor was found to be significant. Adding the ‘frustration relatedness in life’ and ‘frustration relatedness in study’ separately to Model 1 revealed for both significant results: frustration relatedness in life: (*b* = −0.31, *t*(137) = −3.32, *p* < 0.05) and ‘frustration of relatedness in study’ (*b* = −0.22, *t*(142) = −3.48, *p* < 0.05). The more students feel frustrated in their need for relatedness in life, the lesser their academic engagement, with −0.31 per unit increase. The more students feel frustrated in their need for relatedness in their study, the lesser their academic engagement, with −0.22 per unit increase.

When both relatedness frustration scales were put together in Model 2 (see Table 4), they remained significant predictors of academic engagement (frustration of relatedness life: (*b* = −0.21, *t*(136) = −2.10 *p* < 0.05) and frustration of relatedness study: (*b* = −0.16, *t*(136) = −2.19 *p* < 0.05)). The satisfaction scales were also significant positive predictors of academic engagement, but when these scales are added simultaneously with the frustration scales, only the effect of the frustration scales remained significant. Therefore, we only added the frustration scales to Model 1 in the hierarchical regression analysis.

The inclusion of both in the model (Model 2) was found to be a significant improvement compared to Model 1, *F_change_* (2,136) = 8.06, *p* < 0.001 and with this model 19% of the variance in academic engagement could be explained (*R*^2^ = 0.19). The inclusion of the loneliness scales to Model 2 (*cf.* Model 3), did not result in (additional) significant predictors and was not a significant improvement compared to Model 2. This model 3 explained 20% of the variance in academic engagement. A close inspection of this model, however, also revealed that the relatedness frustration scales were not significant anymore either in this model (Model 3).

Table 5 shows that adding ‘social loneliness’ and ‘emotional loneliness’ separately to Model 1 revealed for both significant results: ‘social loneliness’: (*b* = −0.19, *t*(165) = −2.50, *p* < 0.05) and more significant: ‘emotional loneliness’ (*b* = −0.24, *t*(165) = −3.71, *p* < 0.001). The more students feel socially alone, the lesser their academic engagement, with −0.19 per unit increase. The more students feel emotionally alone, the lesser their academic engagement, with −0.24 per unit increase.

When both loneliness scales were put together in Model 2 (see Table 5), only ‘emotional loneliness’ remained a significant predictor of academic engagement (*b* = −0.21, *t*(136) = −2.47 *p* < 0.05). The inclusion of both in the model (Model 2) was a significant improvement compared to Model 1, *F_change_* (2,123) = 3.67, *p* = 0.001 and with this model 17% of the variance in academic engagement could be explained (R^2^ = 0.17). The inclusion of the relatedness scales to Model 2 (*cf.* Model 3), did not result in (additional) significant predictors and a close inspection of this model also revealed that the ‘emotional loneliness’ scale was not significant anymore either in this model.

### 5.3. Which experiences of relatedness and loneliness do students express in relation to their academic engagement?

In this section qualitative data on students’ experiences is examined as to contextualize and clarify our main quantitative findings. Quantitatively, we found that the relatedness and loneliness scales (together with the demographic factors) explained 20% of the variation in academic engagement: the more students felt frustrated in their relatedness (in their school and study context and in their life in general) and the more they felt lonely, the lesser their academic engagement was. Also, we found that the students were, as expected, significantly less satisfied and more frustrated in their need for relatedness in the academic context compared to their need for relatedness related to life in general during COVID times. It is uncertain, however, whether COVID-19 lockdown measures alone caused the “relatedness” difference between general life and the academic context, or if other factors played a role. Using qualitative data, we explored to what extent and how these outcomes might be actually related to the COVID-19 lockdown. The qualitative results pertain to both our key quantitative concepts and relationships as well as to the inductively derived new themes around ‘student relatedness regarding peers versus faculty’ and ‘the academic social (online) context’^.^ The quotes are from various respondents unless specified otherwise.

#### 5.3.1. Relatedness in study and academic engagement

As expected, most students felt less engaged with the academic learning environment than before the COVID-19 lockdown:

I expect the entire upcoming academic year to take place online and this is going to have a huge impact on my motivation and commitment to study. I find it hugely annoying that I don't connect with my fellow students and thus get to know them better. My expectation is that my student life will become a lot less fun because of the measures.

Besides the fact that studying online is less enjoyable according to this account, the study also loses depth and engagement for these students*: ‘Not being able to chat through classes or study together. It gives the study less depth and it becomes less alive’*. In addition, there is also no alternative for students to study together - for free - within the restrictions since COVID-19: *‘Moreover, what I miss most is a place to study for free (like the university library’)*.

For these students, academic engagement, which before COVID-19 appeared to be partly sustained through physically studying together - especially offering a low threshold for supporting each other -, is difficult to maintain due to the higher threshold of getting into touch. Engagement still exists for these students, but it is increasingly influenced by external stress factors such as ‘*time and performance pressure*’ and deadlines than before COVID-19. With lectures and the material less alive due to studying alone at home, the study since COVID-19 has less to do with what these students enjoy:

Before COVID lectures were a way for me to listen and talk to teachers and students in an enjoyable and relaxed way. Now it feels like a dry and boring way to listen to the knowledge the lecturer is droning on, like a radio.

The students further indicate that they miss the spontaneous contacts at the university, such as during the break in the canteen or by passing each other in the building of the university: *‘I missed physical contact with fellow students and the chat at the coffee machine and just walking in spontaneously at the university*.^2^ *The contact is different’*.

For some, this *‘loose’* contact with acquaintances and strangers at university appeared to satisfy their need for relatedness before the COVID-19 lockdown, partly because this form of contact felt *‘natural’*.

Because many of these contacts also occur in passing and when you don't run into each other this can't happen.’

Students indicate that since COVID-19 and the closing of the university, they had to find their own ways to feel related and not frustrated in their relatedness. Previously, this need seemed to be met in part because students run into people at university, with little strain:

‘I miss this terribly, the small conversations, because how I filled my social life before Corona is now quite lonely [sic].’

New students in particular - regardless of age or stage of study - describe how they feel lost at a new university: ‘Not having physical contact, the ability to just walk into someone’s office, the threshold to look up your classmates etc. […] I do feel a bit lost in this study’. The fact that a student, lecturer, or other staff member cannot be addressed spontaneously, raises the threshold of asking for help:

‘Finding your way around a new system is difficult when the system is so far away from you. […] It affects my sense of whether I can do this’.

Another student feels deprived of her own choice of type of education:

‘Already it is said that [the next semester] will also go through Teams. I fear that I will do my master's without meeting a student or teacher, that is really a big fear and is contrary to my choice for this University namely meeting each other [sic].’

According to students, the virtual learning environment has emerged *‘suddenly’* without a replacement for mutual consultation, which occurred at the university before COVID-19:

‘I struggle with the absence of 'real' contact during lectures. Just discussing with neighbors what exactly this text means and how it relates to this other text. Asking a quick question during the lecture doesn't go well either, even if the lecturer invites it. The natural interaction is gone.’

In the above quote, students indicate that the loss of the natural interaction - that before COVID-19 - leads to uncertainty about the quality of their own development within the study.

In addition, students thus mention the lack of a space to go to immerse themselves in the study material. For example, a student in a small, shared apartment states:

‘I have been significantly behind with the material since pretty much the beginning of the master because I don't have time for it, can't find a place to concentrate for it (my room is small and I live in a dorm with all kinds of things to be done due to the move, libraries have very limited space).’

She continues:

‘I then constantly berate myself for failing to make progress in my studies even though I want to. The subject matter does interest me, I just need some extra guidance or structure and a suitable study place to get started’.

She indicates that she lacks guidance and structure, in other words, attention.

The quotes above indicate several issues with studying online. Namely, a higher threshold to ask for clarification and support. Also, online studying does not seem to support the discussing of material between students and teachers, a lack that appears to impede the student’s engagement with the material. First-time students struggle with accustoming to university life. Furthermore, students are increasingly dependent on their own ability to find a proper (free) space to study, as this is no longer provided by their university.

#### 5.3.2. Nuances satisfaction and frustration in academic relatedness

However, also a considerable number of respondents reports a newfound academic engagement due to lockdown measures. Prior to the lockdown, enrolment in higher education meant regular commuting and social interaction. For some students, these peripheral matters appeared to have been taxing. Now that the learning environment was online-only, they noticed that the absence of these peripheral matters provided more *‘mindspace’* and time to engage with the study material:

‘I don’t have to travel by train anymore, which means I can stay in my bed longer! […] I have fewer social contacts, but I actually find that relaxing. I feel much less like I’m being lived and rolling from one social activity into the next.’

These students indicated that their academic engagement has felt more *‘their own’* since COVID-19, as they experience more *‘social peace’* because of the lockdown. According to these students, the ‘*elimination of social obligations’* led to critical self-reflection regarding what really motivated them, what they truly enjoy without external pressure or reward:

‘Since COVID forced everything to stop, I have a kind of clean slate and with the start of my studies I had to make a lot of choices. Now I only do what I really want and/or what makes me feel the pure physical sensation of joy.’

The aforementioned social peace for this respondent led to looking inward: to find a personal drive, not only because the respondent *‘had to’* do certain things. Academic engagement - which before seemed more imposed by social standards - now seems to have shifted more toward a meaningful personal preference. In other words, the source of academic engagement has become less external, rather internal since this student experiences more social peace. In her own words, the source of engagement is even *‘pure physical sensation of joy’*.

This preference for more ‘*alone time’* was not expected. The connection between fewer social stimuli and more academic engagement is also made by other students interviewed, and they themselves see this as *‘unexpected.’* ‘Unexpected,’ because social pursuits and *‘being outside a lot’* were previously taken for granted. They note in their responses that studying - now that they have more time to devote to it - can be made more their own volition than before COVID-19:

‘I have more energy left now that classes are online, I don't have to get up as early and I don't have to be social for an entire school day. […] I also notice now that there is less chaos with traveling back and forth and planning meals/outfits etc. for school, I can keep more of an overview of the study and thus I have more motivation to study.’

The peripheral matters associated with an academic life has proven to take more time and energy than they had realized before COVID-19.

For these students, the fewer forced social stimuli had a positive effect on with whom they do or do not share their time. Where and whom they draw energy from, both for study and in their personal lives, seemed harder to see or feel before COVID-19. During social distancing, these students realized that they were more *‘lived’* by social norms before the COVID-19 restrictions:

‘A lot of obligations around clubs had been canceled which gave me more time to create peace in my life. […] This realization came only during social distancing. As a result, I started to make a more selective choice in who my friends are and who are not. Also, this extra time has caused me to start thinking about what I really want which also creates more clarity in my life.’

This *‘relief’* as a result of the extra time and energy, appears to have a beneficial effect for more students in their personal lives on their ‘*self-preservation*’ and *‘stress-levels’* in personal relationships. These beneficial effects words are linked by these students to greater enjoyment of and thus engagement for their studies:

Since the lockdown, I was suddenly able to remove all sorts of things from my schedule, which I found both unfortunate and a relief. […] That stress has become less now though, I am more in charge of my schedule. I filled the gaps in my schedule with a lot of school efforts, and that’s why I find the education more interesting; I’m more engaged in it and put a lot of love and energy into it.’

These students do indicate - as the above answer illustrates - that it is unfortunate that so many contacts are harder to maintain, but at the same time the *force majeure* of social distancing rules provides a *‘legitimate reason’* to excuse themselves, whereas they could not excuse themselves before without feeling ‘*socially inadequate*’. They write about how they can now interact with people in a way that they themselves support whilst experiencing less pressure.

As mentioned earlier, this more individual and meaningful way of feeling connected seems to have a beneficial effect on academic engagement for these, more (self-proclaimed) introverted students. The latter seems to be based more on what the students themselves want in their lives, and how the study fits into that:

‘[…] I can recognize for myself that this "break" from the daily flow (of impressions/commitments) actually unintentionally helped me a lot in my own process of recognizing what I need. Because I better recognized what I wanted, this study came into view and for now it has turned out to be a good match. A lot has finally fallen into place since years.’

In the above context, a ‘*break*’ is described as a positive aspect of social distancing, while ‘*pause*’ - when discussing frustration of relatedness and social loneliness earlier in this article - was described as an indeterminately negative association with loss of social interactions in daily life. These different voices show how diverse the manifestations of both satisfaction and frustration of relatedness are for each individual.

However, clarifying how it can lead to more academic engagement, the social tranquility, according to these students leads to more concentration for study, which in turn leads to more engagement and enjoyment while mastering the material:

‘I do think I can concentrate much better and have more energy to study because I don't have to travel, I don’t have the stimuli of being on location.’

They cite positive effects such as *‘more time for introspection’,* making choices in study and peripheral matters regarding academic life more well-considered. It was unexpected that less social contact following COVID-19 restrictions could correlate with more academic engagement, but these students unmistakably describe that social rest and home study can lead to a more sustainable form of academic engagement:

‘I personally really like taking college online because I can concentrate better and have more energy for other things. I experience less stress because I am always on time and I have attended all lectures so far while I normally skipped almost half of them.’

So, for some students, having time and attention to spare appears to be conducive to their study progress and the enjoyment they get from studying. They also reported to be no longer *‘distracted by fellow students’* during lectures and pressured to be socially well-liked in the study group and by teachers.

#### 5.3.3. Differentiation relatedness (in)between student cohorts and faculty

The qualitative results also supplemented the quantitative results with regard to making a difference between relatedness within student group and between students and members of faculty.

As stated earlier in this article, the students sometimes feel related within their cohort group, but no longer to the teachers or any faculty member. ‘*[A] lecturer droning on like a radio’,* generally does not increase the bond between teacher and subjects. And while there is a general understanding and empathy towards the teachers in the qualitative data (*‘they are trying their best’*), the online lectures seem to create a chasm between the teacher and the student. *‘Joy’* in the interaction between student and teacher is lost in the online learning environment.

However, the needs of relatedness also differ in stages of life. Whereas an 18-year-old student who just started higher education needs more guidance from faculty members to engage academically, an older student with more lived experience and a family at home might consider this guidance superfluous. Furthermore, a new student might be more intent on making friends during their college years, whereas an older student has different priorities.

It turns out that not only roommates can play an important role in the satisfaction of relatedness and feeling less lonely, but also loved ones in certain stage of life, such as having a family of their own: ‘*I do think it is a great advantage that I live with 3 children and husband at home and not alone. Then I might have enjoyed it less*’.

The above indicates that even though students may feel less related and more (socially) lonely regarding their learning environment compared to the situation before COVID-19, they also feel unexpectedly enabled to find relatedness in their close surroundings.

#### 5.3.4. Academic community exclusion and loneliness

Loneliness – in this article – is defined as relatedness to others that falls short in comparison to what is *expected* of feeling related. Even though some students seek creative solutions to this resulting loneliness, it still does not satisfy their need of ‘*being part of a greater whole*’, i.e., being part of the university that provided space for community before COVID-19. The lack of community emerges in the responses: *‘I feel that it is mainly the indirect effects that I am slowly noticing. A background sense of commonality that I normally experienced within the study.’*

The feeling of belonging to a community appears to be important to feel related, especially the aspect of *‘being important’* to a group. In other words, it is important to one’s sense of belonging, that not only other individuals, but also a community or organization cares about them. Those students who report feeling alone since COVID-19 mostly attribute their loneliness to the lack of help and explanation from both the administration and the university:

‘I especially feel a lot of sadness because of the cold attitude the university has toward me during Corona.’

The following section illustrates the disappointment of students in the attitude of the university and its staff since COVID-19. One student clearly explains in his own words where the frustration lies, despite the benefits since COVID-19:

‘When this school year [September 2020] started again after the summer vacation, I had expected and hoped to be briefly addressed by our teacher or someone else from the university, welcomed to the final year and some information, perspectives, time to share things with each other. However, this was not the case at all which made the start of the year and of my last school year very strange. From one day to the next we started class immediately as if nothing had changed. I find that I need more updates, information and reassurance and support from the university.’

This student - with some others - expressed the feeling that they do not matter as a group or community; both in the eyes of the government and in the eyes of their university. They apparently do not feel ‘*recognized*’ and acknowledged in their needs during the lockdown; in these responses this is mainly because since COVID-19, ‘*[the university] does not help me with anything anymore*’. Some indicate feeling anger and helplessness because they had expected to be helped during such a situation. Their experiences at the university before COVID-19 made them believe that they would be recognized as an affected group. But since COVID-19, they feel unimportant and even feel *‘stupid’* for expecting otherwise:

‘I feel very stupid because the university does not offer help during Corona times on the grounds that they themselves [suffer] from it, as if I did not. Very sad.’

These students feel *‘not recognized’, ‘not heard’*, and thus dismissed as unimportant ‘*collateral damage*’ in a crisis. They indicate in their responses that they do not feel ‘*engaged*’ because of this, as if both the administration and the university do not seem to *‘care’* about them since COVID-19:

I don't feel heard by the university or faculty. And I don't feel like anyone wants to engage in conversation.

Moreover, some of the students say that their opinions have not been asked for regarding education since COVID-19, by both the administration and the university: *‘Still I run into things that I want to share on and with university. It’s important to experience support from organizations that you are part of (which I do not experience at all)’.*

Thus, it is not just experiencing no support *‘from organizations you are part of’* that causes students to feel not cared for since COVID-19, but also the perceived hurdle for sharing their opinion with others – being heard by organizations and therefore feeling important enough to receive *‘accountability’* and *‘explanations’.* This creates a picture in which there is a gap in the learning environment between on the one hand the students and on the other hand the university and the government. These students express disappointment in their expectation that the university would be more on their side since COVID-19:

‘Above all, I think that you have to figure it out for yourself and that this is secretly even expected of the university students unconsciously. […] No one would be so quick to call if you don't show up in the online lectures.’

The above quote expresses an experience of ‘*feeling invisible’*. Thus, the feeling that it would not matter to the community - of which they thought they were a part - whether they showed up or not has been reinforced for some of the students interviewed since COVID-19.

## 6. Discussion and conclusions

### 6.1. Conclusion and link with literature

In this study, higher education students’ academic engagement during COVID-19 times was addressed and related to experiences of loneliness and relatedness (in life and in school social study context), applying a mixed-methods approach. Previous research showed that higher education struggled to keep students on task in the mandated virtual classrooms during *social distancing* as mandated due to COVID-19 (Kiltz et al., 2023), that students felt (more) lonely (Benke et al., 2020; DUWO and Youngworks, 2020; Marchini et al., 2020; Labrague et al., 2021; Rania et al., 2022; Kiltz et al., 2023), and that mental health problems among students increased during the lockdown (Dadhich et al., 2022; Rania et al., 2022; Kiltz et al., 2023).

When we looked at the impact of relatedness and loneliness on academic engagement, quantitative results were as expected. Namely, that the frustration of relatedness in study and life correlated significantly negative with academic engagement. Social and emotional loneliness also proved to be significant predictors of academic engagement in another series. The loneliness and relatedness scales were, however, no longer significant predictors when added to the other.

In addition, the qualitative responses complement and provide insight into how much of the quantitative results can be attributed to the actual COVID-19 lockdown. Respondents reported positive effects of increased solitude, positing unexpected “relief” of removed social obligations due to the mandated *social distancing*. This *force majeure* led a considerable number of students to re-evaluate their career in higher education and taking conscious steps, which led to their newfound academic engagement. Some students say they are more interested and committed to their studies because they experience less stress from, for example, constantly “having” to do social interactions at university. The “force majeure” of COVID-19 gives these students an excuse to “recharge” for a while without having to cancel appointments.

These accounts are in line with the literature which suggests that being physically alone (being allowed to be alone) can have positive effects on well-being (De Jong-Gierveld and Havens, 2004; De Jong-Gierveld et al., 2006; Cacioppo and Patrick, 2009; Nguyen et al., 2021). However, these surprisingly positive narratives do not take away from the fact that overall, the qualitative results support the quantitative results, which indicate more frustration in relatedness and more loneliness. As expected following COVID-19 research (Benke et al., 2020; DUWO and Youngworks, 2020; Marchini et al., 2020; Labrague et al., 2021; Kiltz et al., 2023), students’ own articulations shine a personal light on why social distancing proves unfavorable for students. For most, their personal interest and commitment suffers, and they worry about how long this will last. They also worry about how long they will be able to sustain online education, many indicating they want to be *‘done as soon as possible’* and that they do not expect much more from university or their student life. A surprising number of students indicated that they felt let down by the university by the lack of communication and that this feeling of abandonment led to them falling behind with their studies.

Results show that students’ academic engagement suffers from the loss of a shared physical space and growing uncertainty for most students (Kiltz et al., 2023), but also that for some students the loss of social obligations due to force majeure has been a blessing in disguise. That it is not so straightforward or purely negative is particularly clear from the qualitative results. Quantitative results show – in line with SDT (Vansteenkiste et al., 2020, p. 1) that students feel little support in their need to “care” about other members in their social school study context. Moreover, the frustration of relatedness (since this need works both ways) also indicates a lack of feeling “cared for” by the social context (*ibid.*), in this case significantly more in the study context than in life. This difference may exist because the need for relatedness in the general “life” domain may be compensated by, for example, family, colleagues, housemates and/or mini-interactions with passers-by. Social mini-interactions, for example, appear to be salient in satisfying the need for relatedness and feeling less lonely and/or frustrated in the need for relatedness (Cacioppo and Patrick, 2009; Sandstrom and Dunn, 2013, 2014; Hertz, 2021).

The qualitative results of this study indicate this difference: namely, that mainly students with families or roommates feel more connected in general than with their fellow students and teachers. As they are forced to study and work at home due to the COVID-19 measures, they report experiencing more attention and time for their loved ones. They had less time for this before 12 March 2020. This contact is something that appears to be a pleasant, unexpected side effect of social distancing. Contact with fellow students is harder to maintain online, according to respondents. They experience little opportunity or alternatives for contact between students and teachers. This lack is in part in contrast to the new connectedness they experience with people who are “trapped” with them at home (e.g., family, housemates, etc.).

Despite positive experiences of connectedness outside the study context, it is important to look at the area in which this is frustrated. According to the SDT, the effect of not feeling connected to the social context of the learning environment is ‘a sense of social alienation, exclusion, and loneliness’ (Vansteenkiste et al., 2020, p. 1).

This did not show up in the quantitative results however, which may be due to the sensitivity of questions about feeling excluded and rejected for respondents (Cacioppo and Patrick, 2009). “Admitting” experiences or situations such as ‘loneliness’ can cause embarrassment and discomfort. In addition, qualitative results suggest that within the frame of reference of social distancing, people have come to value social connectedness differently.

Nonetheless, qualitative data of this study shows that having an intense relationship (romantic or with family) helps against loneliness, including social loneliness. This finding is consistent with recent research focusing on buffers against the psychological harmful effects of social distancing (Li and Xu, 2020). Even if students notice that they have less contact with their peers and teachers, the fact that these students either have a family or a partner provides a “barrier” against the negative effects of loneliness.

### 6.2. Limitations and recommendations

#### 6.2.1. Limitations

In this study, psychological effects of social distancing were measured after 6 months of lockdown. An important question is to what extent psychological effects of social distancing are already measurable after 6 months. As Sher (2020) indicates, psychological effects of a crisis are not always immediately observable. In addition, even though this study indicates a trend of more frustration and less fulfillment, a full longitudinal design with multiple points of measurement would be needed to assess a trend and possible changes in the future.

Another limitation concerns the set-up of our online survey questionnaire in which the standardized questions on relatedness and loneliness preceded the open-ended questions. In their open answers, the respondents used similar words to the words used in the closed-ended part of the survey. They thus may have ‘primed’ students to think about concepts such as motivation and loneliness in their open responses (Gobo and Mauceri, 2014). An alternative approach of data collection could then have been to send out two separate questionnaires: a standardized one and a question-list with open questions only. This might also have ensured fewer *missings* at the end of the survey, which was perceived as ‘too long’ by some respondents. However, while this dual questionnaire excludes priming effects, it also increases the risk of additional unit non-response since respondents might omit one of both questionnaires.

On a substantive level, a relevant limitation may concern the conceptualization of ‘relatedness’, which is measured as an overarching concept in our study. The qualitative results, however, suggest that distinguishing ‘relatedness’ for different target groups, like faculty staff and students might be more appropriate (following Beachboard et al., 2011), as some might feel related to student peers whilst not related to faculty members (or the other way around). Additionally, it remains conjecture in the quantitative results about what is considered ‘relatedness’ in a crisis (Pan et al., 2021).

Furthermore, we did not inquire about students’ personality traits, so we could not explore whether extraversion or introversion could explain the differences in experiencing the benefits of solitude.

Another limitation is that we focused solely on one, rather small university offering studies in the domains of humanities and social sciences. This may compromise the generalizability of the findings to students from larger universities and enrolled in other studies. Also: As the university is known for personal interaction, its students may have a stronger inclination to value shared personal space compared to students at other institutions.

#### 6.2.2. Recommendations for future research and educational policy

In general, despite the mentioned limitations, the study shows the value and richness of examining the impact of relatedness and loneliness on academic engagement using a mixed-methods approach. Furthermore, the findings of the study make a clear contribution to the knowledge base bringing in additional insights and nuances. We recommend therefore to make use of this approach more often when studying comparable topics.

Now the question is what can be learned from the results: how could a next occasion of social distancing be addressed in higher education and in educational policy? To start with, both our quantitative and qualitative results indicate that a physically shared space is generally preferred for students. But of course, given possible contamination risks the use of shared spaces might be necessarily limited (for example to outside locations, or small study groups). In addition, this study also brought to light that the need for relatedness and the impact on students’ academic engagement differs for every individual. Despite most students struggling to stay on task during the lockdown, there were also students thriving academically. Thus, a recommendation (for research and policy) would be to further explore what relevant types of students can be distinguished and what they would need in terms of support during their academic career.

Another, major recommendation relates to the outcome that many students were surprised that this survey - after 6 months of online education - was the first time anyone had asked them extensively about their experiences. This brings us to the advice for higher education to start interaction with students about the situation earlier. Communication, despite bearing negative news, makes members of a community feel “worthy of an explanation” (Cacioppo and Patrick, 2009). Most students felt lonely because of the discrepancy between what they thought the university would do in such a crisis, and what actually happened: less communication. This means that if an institute or organization takes its’ members along in the situation by acknowledging their struggle, the members will feel less “left in the dark” and more “important.” This, according to our findings, would lead to a desirable learning environment with supported academic engagement. The connection between feeling unimportant to the community and a loss of interest and persistence in higher education is worth looking into. Possibly, students tend to feel less engaged with their academic context if they *sense* that the academic context (in this case: the university) does not engage with its students. Therefore, we particularly stress the importance of communication since a lack of it can lead to an unintended breach of comfort and trust between members of a community (such as higher education) and the facilitating members (such as the faculty staff).

In the qualitative results, there are numerous complaints about the university resulting from the students’ frustration in not having been asked before. Students also indicate that the silence and lack of clarity are understandable as the university knew as little about the duration and course of action as they did. This pandemic meant that not only students, but also policymakers and lecturers had to switch very suddenly (Daniel, 2020), without having time for any preparation like taking courses on “online lecturing.” Moreover, nobody knew that this was going to take at least a year. Now, however, we know more about the effects of ‘social distancing’ on students thanks to studies like this one on the impact of COVID-19 measures. In the future, researchers, educators, and policy makers can make use of such results while responding to similar measures no one is prepared for. The ‘COVID-19 lockdown’ worked as an unintended natural experiment, which, besides being very challenging, proved instructive for the future.

## Data availability statement

The raw data supporting the conclusions of this article will be made available by the authors, without undue reservation.

## Ethics statement

The studies involving humans were approved by Ethics Committee University for Humanistics Studies. The studies were conducted in accordance with the local legislation and institutional requirements. The participants provided their written informed consent to participate in this study.

## Author contributions

LH and M-CO did the conceptualization and designed the study. M-CO was in charge of the data collection procedure and supervised the data analysis. LH did the data collection and data analysis and wrote an initial draft of the manuscript. WV reinstalled the qualitative and mixed-methods conception of the study. LH, M-CO, and WV all contributed to the final profiling and writing of the manuscript, commended on previous versions of the manuscript and read and approved the final manuscript.

## Conflict of interest

The authors declare that the research was conducted in the absence of any commercial or financial relationships that could be construed as a potential conflict of interest.

## Publisher’s note

All claims expressed in this article are solely those of the authors and do not necessarily represent those of their affiliated organizations, or those of the publisher, the editors and the reviewers. Any product that may be evaluated in this article, or claim that may be made by its manufacturer, is not guaranteed or endorsed by the publisher.

## Footnotes

^1^The relatively low Cronbach value is common in the case of scales with fewer than 10 items (e.g., 0.5; Pallant, 2020). Therefore, following up on Briggs and Cheek’s (1986) suggestion in cases like these, we also looked at the mean inter-item correlation for these two items, which was 0.410. Clark and Watson (1995) mention an optimal range between 0.15 and 0.50. McDonald’s ɷ could not be calculated for these scales since each scale had only two indicators.

^2^For reasons of privacy and research purposes, the respondent’s mentioning of their specific university has been replaced by ‘university’.
