# Supplementary material for: Students’ academic engagement during COVID-19 times: a mixed-methods study into relatedness and loneliness during the pandemic
Source: Front Psychol. 2023 Sep 8;14:1221003. doi: 10.3389/fpsyg.2023.1221003 (PMC10514504; doi:10.3389/fpsyg.2023.1221003)
Supplement: Supplementary file 1 [file Data_Sheet_1.docx]

| *Table 6: Nested crosstabulation participants – study phase* | | | | | | | |
| --- | --- | --- | --- | --- | --- | --- | --- |
|  | | | | Study phase | | | |
|  |  |  |  | Bachelor | Pre-master | Master | Total^*^ |
|  |  |  |  | % | % | % | % |
| Gender | Male | Age categories | Missing |  |  | 0.48 | 0.48 |
|  |  |  | 18 - 25 | 10.14 |  | 1.45 | 11.59 |
|  |  |  | 26 - 33 | 0.48 |  | 2.90 | 3.38 |
|  |  |  | 34 and older |  | 0.48 | 1.93 | 2.42 |
|  | Female | Age categories | Missing |  |  | 1.45 | 1.45 |
|  |  |  | 18 - 25 | 30.92 | 3.86 | 14.49 | 49.28 |
|  |  |  | 26 - 33 | 2.90 | 4.83 | 10.63 | 18.36 |
|  |  |  | 34 and older |  | 2.90 | 10.14 | 13.04 |

**Supplementary Material: Tables and additional population data for participants**

| *Table 7: Nested crosstabulation participants – living situation* | | | | | | |
| --- | --- | --- | --- | --- | --- | --- |
|  | | | | Living situation | | |
|  |  |  |  | Cohabiting | Living alone | Total^*^ |
|  |  |  |  | % | % | % |
| Gender | Male | Age categories | Missing | 0.99 |  | 0.99 |
|  |  |  | 18 - 25 | 11.33 | 0.49 | 11.82 |
|  |  |  | 26 - 33 | 2.46 | 0.99 | 3.45 |
|  |  |  | 34 and older | 0.99 | 1.97 | 2.96 |
|  | Female | Age categories | Missing | 0.99 | 0.49 | 1.48 |
|  |  |  | 18 - 25 | 40.39 | 8.37 | 48.77 |
|  |  |  | 26 - 33 | 14.29 | 3.94 | 18.23 |
|  |  |  | 34 and older | 11.33 | 0.99 | 12.32 |

# Additional group information of respondents

^*^ *N = 207*

^*^ *N = 203*

# Additional data on student population

According to the student population (N = 611) at the time the study took place, data retrieved from the university shows that 56.5% of the respondents were 18-25 years old, 27.2% between 26-33 years, 5.2% between 34-41, 3.9% between 42-49, 4.9% between 50-57 and 2.1% between 58-65 years old (see Table 8). According to student population (N = 611), 76.4% were female students (N = 611) (see Table 9).

According to student population (N = 611), 42.6% were Bachelor students, 440% Master students and 13.4% Pre-master students (N = 611) (see table 10).

The university has no information considering the ‘living situation’ of the 611 students enrolled during the data collection for this research.

| *Table 8: Distribution of the student population by age groups* | | | | | |  |
| --- | --- | --- | --- | --- | --- | --- |
|  | | Frequency | Percent | Valid Percent | Cumulative Percent |  |
| Valid | 18-25 | 345 | 56.5 | 56.6 | 56.6 |  |
|  | 26-33 | 166 | 27.2 | 27.2 | 83.8 |  |
|  | 34-41 | 32 | 5.2 | 5.2 | 89.0 |  |
|  | 42-49 | 24 | 3.9 | 3.9 | 93.0 |  |
|  | 50-57 | 30 | 4.9 | 4.9 | 97.9 |  |
|  | 58-65 | 13 | 2.1 | 2.1 | 100.0 |  |
|  | Total | 610 | 99.8 | 100.0 |  |  |
| Missing | System | 1 | 0.2 |  |  |  |
| Total | | 611 | 100.0 |  |  |  |

| *Table 9: Distribution of the student population by gender groups* | | | | | |  |
| --- | --- | --- | --- | --- | --- | --- |
|  | | Frequency | Percent | Valid Percent | Cumulative Percent |  |
| Valid | Male | 144 | 23.6 | 23.6 | 23.6 |  |
|  | Female | 467 | 76.4 | 76.4 | 100.0 |  |
|  | Total | 611 | 100.0 | 100.0 |  |  |

| *Table 10: Distribution of the student population by study phase groups* | | | | | |  |
| --- | --- | --- | --- | --- | --- | --- |
|  | | Frequency | Percent | Valid Percent | Cumulative Percent |  |
| Valid | Bachelor | 260 | 42.6 | 42.6 | 42.6 |  |
|  | Master | 269 | 44.0 | 44.0 | 86.6 |  |
|  | Pre-master | 82 | 13.4 | 13.4 | 100.0 |  |
|  | Total | 611 | 100.0 | 100.0 |  |  |
